# The IoRT-in-Hand: Tele-Robotic Echography and Digital Twins on Mobile Devices

**DOI:** 10.3390/s25164972

**Published:** 2025-08-11

**Authors:** Juan Bravo-Arrabal, Zhuoqi Cheng, J. J. Fernández-Lozano, Jose Antonio Gomez-Ruiz, Christian Schlette, Thiusius Rajeeth Savarimuthu, Anthony Mandow, Alfonso García-Cerezo

**Affiliations:** 1 Institute for Mechatronics Engineering and Cyber-Physical Systems (IMECH.UMA), University of Malaga, 29071 Malaga, Spain; 2 Mærsk Mc-Kinney Møller Instituttet, University of Southern Denmark, 5230 Odense, Denmark; zch@mmmi.sdu.dk (Z.C.); jfl@uma.es (J.J.F.-L.); janto@uma.es (J.A.G.-R.); chsch@mmmi.sdu.dk (C.S.); trs@mmmi.sdu.dk (T.R.S.); amandow@uma.es (A.M.); ajgarcia@uma.es (A.G.-C.)

**Keywords:** Android, cloud robotics, Digital Twin, medical instruments, teleoperation

## Abstract

The integration of robotics and mobile networks (5G/6G) through the Internet of Robotic Things (IoRT) is revolutionizing telemedicine, enabling remote physician participation in scenarios where specialists are scarce, where there is a high risk to them, such as in conflicts or natural disasters, or where access to a medical facility is not possible. Nevertheless, touching a human safely with a robotic arm in non-engineered or even out-of-hospital environments presents substantial challenges. This article presents a novel IoRT approach for healthcare in or from remote areas, enabling interaction between a specialist’s hand and a robotic hand. We introduce the IoRT-in-hand: a smart, lightweight end-effector that extends the specialist’s hand, integrating a medical instrument, an RGB camera with servos, a force/torque sensor, and a mini-PC with Internet connectivity. Additionally, we propose an open-source Android app combining MQTT and ROS for real-time remote manipulation, alongside an Edge–Cloud architecture that links the physical robot with its Digital Twin (DT), enabling precise control and 3D visual feedback of the robot’s environment. A proof of concept is presented for the proposed tele-robotic system, using a 6-DOF manipulator with the IoRT-in-hand to perform an ultrasound scan. Teleoperation was conducted over 2300 km via a 5G NSA network on the operator side and a wired network in a laboratory on the robot side. Performance was assessed through human subject feedback, sensory data, and latency measurements, demonstrating the system’s potential for remote healthcare and emergency applications. The source code and CAD models of the IoRT-in-hand prototype are publicly available in an open-access repository to encourage reproducibility and facilitate further developments in robotic telemedicine.

## 1. Introduction

The COVID-19 crisis served as a catalyst for the rapid advancement of Internet of Things (IoT)-based healthcare technologies, enabling early infection detection, remote patient monitoring, and more efficient treatment and resource management. Examples include connected thermometers, pulse oximeters, smart medical devices, and Bluetooth-enabled contact tracing systems. In the aftermath, billions of IoT devices have been deployed to support real-time hospital monitoring [1] and decentralized clinical trials, facilitating remote patient tracking and diagnostics.

Attaching heterogeneous tools such as grippers, cameras, test instruments, or force sensors to the end-effector (EE) is a common practice in manipulation robotics. In this context, authors in [2,3] developed a robot for COVID-19 oropharyngeal-swab sampling, integrating a Micro-Pneumatic Actuator (MPA) on the EE.

However, a standardized solution for the integration and control of such tools across most Compact Robotic Arms (CRAs) in remote scenarios is still lacking. This gap is particularly critical in Search and Rescue (SAR) scenarios, where mobile manipulators are essential for performing complex tasks in hazardous environments, reducing the need for direct human intervention and minimizing risk. Examples include placing sensors on victims for real-time monitoring [4] or performing medical procedures in the absence of a specialized physician [5,6], especially remote areas. Telemedicine assumes a critical role in this context because first responders encounter difficulties in making quick diagnostics post-disaster, mainly attributed to the time needed for operational readiness [7,8] and search and evacuation of victims. In addition, the surviving or unharmed local population often conducts most of the initial exploration in the immediate aftermath of the event, focusing on search rather than rescue [9]. Given the logistical challenges and the urgency of providing aid, Uncrewed Aerial Vehicles (UAVs) are commonly used to deliver packages containing essential supplies to isolated populations [10], such as water, food, clothing, communication devices, and medical essentials, often accompanied by instructions. Most medium UAVs used in these applications can carry and deliver a payload of 3 kg [11,12], which may be a limitation depending on the needs. However, while UAVs primarily serve as delivery systems for instrumentation and operational instructions, it is the Uncrewed Ground Vehicles (UGVs), acting as mobile manipulators, that ultimately execute complex tasks in the field, which could include some medical procedures.

Therefore, developing a plug-and-play mechanism that enables rapid tool attachment, real-time connectivity, and compatibility with different robotic arms remains an unresolved challenge in disaster scenarios. The main obstacles include the following:

- Designing a plug-and-play end-effector mechanism that ensures universal compatibility with any CRA.
- Enabling the end-effector to automatically establish an Internet connection upon being plugged in.
- Implementing real-time monitoring of the manipulator’s environment while ensuring safe robot control, minimizing risks during human interaction.

Regardless of whether the remote scenario involves the teleoperator’s location or the environmental conditions where the robotic manipulator is deployed (e.g., in SAR operations or isolated communities), once the medical tool is mounted on the robot’s EE, two critical challenges arise.

First, it is essential to select an appropriate teleoperation interface and ensure a reliable, low-latency communication link between the operator’s device and the robotic system. Any delay or loss of information can compromise the precision required for medical procedures.

Second, the system must provide the teleoperator with a rich and accurate perception of the robot’s surroundings. In particular, visual feedback should ideally include 3D perspective or depth information, enabling the operator to understand the spatial relationship between the tool, the patient, and nearby obstacles. This situational awareness is critical to perform safe and effective manipulations in complex or unstructured environments.

1. **Choosing a suitable human–robot interface for medical teleoperation.**
  A variety of manual control interfaces, ranging from traditional input devices such as mice, keyboards, and joysticks, to more advanced options like haptic controllers, have been employed in telemanipulation tasks involving robotic arms [13]. Beyond enabling basic control, these interfaces increasingly aim to fuse physical and virtual environments, allowing the teleoperator to receive multi-modal feedback such as force cues, visual overlays, or spatial guidance, directly tied to the robot’s state or its surrounding environment. Nevertheless, in medical teleoperation scenarios, the operator is typically a healthcare professional with limited technical expertise and no direct engineering support, often working in a non-specialized or resource-constrained setting. To promote usability and broader adoption, the control interface should be portable, maintain reliable connectivity, and be based on familiar consumer technologies such as touchscreen tablets. These platforms not only reduce the learning curve but also offer a direct and intuitive interaction model.
  However, not all touch-based interactions are equally sensitive to latency [14]. In particular, dragging gestures, which involve continuous motion, are significantly more sensitive than tapping, with just-noticeable difference thresholds as low as 11 ms in direct-touch settings (where the user touches the screen they are observing), compared to 69 ms for tapping in the same context. In indirect-touch configurations, where the control surface is physically separate from the display (e.g., a touchpad controlling a remote screen), the thresholds rise to 55 ms for dragging and 96 ms for tapping. These findings emphasize the importance of minimizing latency, especially when using direct-touch devices for continuous teleoperation tasks such as guiding a robotic arm or navigating a camera feed.
  In contrast to haptic interfaces, which are typically expensive, technically demanding, and poorly suited for mobile or field applications, touch-based systems offer a more practical and scalable solution. While haptics offer valuable force feedback, their remote implementation is challenged by transmission delays and potential instability. Authors in [15] employ Omega.7 haptic devices controlled via foot interfaces to allow a single surgeon to operate two robotic assistant arms during laparoscopic procedures. The system requires a dedicated physical setup, is not portable, and demands the surgeon’s physical presence in the operating room. In contrast, tablets and other consumer-grade touch devices support fast, reliable interaction and are well-suited for real-world medical teleoperation, particularly when coupled with rich visual feedback and low-latency communication.
  The Internet of Robotic Things (IoRT) framework supports these requirements by allowing operators to use lightweight user interfaces on common devices (e.g., tablets or laptops) that connect to cloud services. Meanwhile, sensing, actuation, and local computation are delegated to Edge devices located near the robot. This architecture enables real-time data fusion, patient monitoring, and remote execution of physical actions in a scalable and interoperable manner [16].
  While many of the reviewed approaches rely on complex or immersive hardware, it is important to recognize that teleoperation through more accessible interfaces, such as touchscreen-based devices, can also yield high-quality datasets for Learning from Demonstration (LfD) [17]. The key challenge becomes how to embed effective multi-modal human–robot interaction (HRI) into these simpler platforms, especially in remote or resource-constrained environments where deploying sophisticated systems is not feasible.
2. **Perception and Feedback for Remote Workspace Awareness.**
  The practical implementation of digital medicine increasingly depends on the integration of Digital Twins (DTs) and the IoRT [18]. A DT goes beyond being a simple virtual replica. It establishes continuous, bidirectional communication with its physical counterpart, enabling real-time data collection, analysis, and monitoring. This dynamic virtual model supports simulation-based optimization and significantly enhances robotic performance in real environments. A robot such as a CRA used in remote surgery can be paired with a DT that visualizes its real-time state and position, simulates trajectories before execution, and detects anomalies or potential collisions. This virtual counterpart supports the human operator by enabling safer and more informed decision-making during teleoperation.
  DTs also provide valuable insights for a range of healthcare applications and services [19,20,21], and their capabilities can be further expanded through Augmented Reality (AR), which improves visualization and user interaction. In telemedicine, DT-based shared control can deliver essential safety mechanisms, such as mitigating the effects of network delays and offering virtual guidance via virtual fixtures [22,23]. Additionally, AR has been explored to boost the operator’s spatial understanding of the robot’s workspace [24], though its effectiveness is strongly tied to the accuracy and fidelity of the DT’s representation.

The integration of DTs and the IoRT in healthcare has led to the emergence of diverse communication protocols between physical and virtual systems, as well as among multiple robotic agents. Although frameworks such as the Robot Operating System (ROS) offer standardized middleware for robotic communication, they fall short in addressing the complexities of seamless integration between DTs and IoT devices. A unified framework that ensures efficient data acquisition, processing, and cybersecurity is still missing. Moreover, there is a need for well-documented case studies to investigate these challenges and identify viable, real-world solutions.

In high-risk environments, teleoperated robots require more than just pre-programmed routines and data analysis. Also, they benefit from strategies that enhance operator effectiveness. DT servers support imitation learning, enabling robots to observe expert human operators and autonomously replicate tasks. This scalable infrastructure can coordinate multiple robots within a network, increasing responsiveness in time-sensitive or critical situations.

This study introduces a tele-robotic system that combines IoT and DT technologies to enable remote ultrasound examinations conducted by a medical specialist using a mobile device over cellular networks. To ensure safe and effective operation, both the physical workspace and its virtual representation (including a DT) are accessible. Key data such as contact forces and the patient’s body position is continuously captured and transmitted over a high-bandwidth, low-latency network, providing real-time feedback to the operator and, when needed, to supporting personnel.

**Figure 1:**
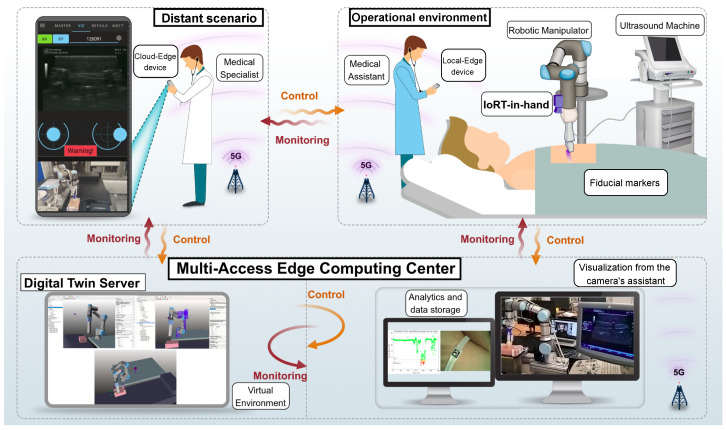
IoT application for ultrasound scanning in a geographically isolated health center. The patient needs to be diagnosed by a medical specialist who is not available in the area. A tele-robotic echography is performed by the specialist using the proposed open-source app to control the robot while performing the remote diagnosis. A view of both the virtual and real environments is available on the smartphone screen since the smartphone can access to the DT hosted on an MEC center and to the images provided by the RGB camera on the IoRT-in-hand. Analytics and data storage are implemented in the Cloud. A high-bandwidth and low-latency network is chosen and setup for the architecture.

The proposed system integrates several interconnected components specifically designed for a telemedicine use case: remote ultrasound scanning (see Figure 1). This work addresses a gap in the current literature and offers the following key contributions:

- Introduction of an IoRT-in-hand device that integrates a compact RGB camera with an embedded torch, two servos for camera orientation, an ultrasound scanner, a force/torque sensor to measure interaction with the patient’s body, and a mini-PC for processing and transmitting data from these IoT Edge components.
- Deployment of a DT hosted on a cloud server running VEROSIM, a 3D simulation platform tailored for advanced robotic applications [25]. This DT provides the operator with a real-time 3D view of the patient within the workspace, complementing the RGB video feed. The virtual environment allows for reviewing and validating motion commands prior to execution on the physical system, thereby improving safety and precision.
- Development of an open-source Android application that integrates ROS nodes and Message Queue Telemetry Transport (MQTT) clients. This app enables control of both the physical robotic manipulator and its DT within VEROSIM via MQTT, in line with the programming approach proposed in [26].
- Implementation of an extended Internet of Cooperative Agents (IoCA) architecture [27] to interconnect all system components in a cohesive and scalable manner.
- Execution of a series of real-world experiments to validate the IoRT-in-hand for tele-robotic ultrasound using a smartphone. Initial tests were conducted in local mode to characterize system behavior, followed by remote teleoperation trials between Malaga (Spain) and Odense (Denmark), where the DT server was hosted.

To the best knowledge of the authors, no previous works have addressed these issues.

The rest of the article is structured as follows: Section 2 reviews related work. Section 3 describes the proposed tele-robotic mobile-based system, followed by Section 4, which details the technical implementation. Section 5 discusses the experimental results obtained between Spain and Denmark. Finally, Section 6 shows the lessons learned, and Section 7 presents the conclusions and future work.

## 2. Related Work

Robotic systems for telemedicine have gained increasing relevance in scenarios where access to specialized care is limited. This section reviews related work in three key areas: remote control of medical manipulators, the design and integration of EE tools, and the communication architectures supporting remote operation.

### 2.1. Remote Control Interfaces for Robotic Manipulators in Telemedicine

In 2001, the first transatlantic robot-assisted telesurgery was performed, involving a laparoscopic cholecystectomy conducted from New York on a patient in Strasbourg, using the ZEUS robotic surgical system (Computer Motion, Sunnyvale, CA, USA) [28]. The surgical and patient stations were connected via a dedicated 10 Mbps optical fiber link utilizing Asynchronous Transfer Mode (ATM) technology, resulting in a total latency of approximately 155 ms. This delay falls well within the range tolerated by the human visual–motor system, typically between 200 and 300 ms. A recent systematic review of clinical telesurgery applications reports latency values between 28 ms and 280 ms, with delays under this range generally considered safe for remote operation [29]. The latency achieved in this early demonstration enabled both successful animal trials and a complication-free human procedure, illustrating the technical and clinical viability of long-distance robotic surgery.

The leader–follower methodology, in which the operator’s motions are directly mirrored by the remote robotic system in real time, has been widely adopted in telemedicine applications due to its intuitive and responsive control scheme. In [30], a medical doctor (MD) remotely performed auscultation, palpation, and ultrasound using a customized end-effector developed by Franka Emika, highlighting the potential of precise, task-specific robotic tools for remote diagnostics.

Beyond leader–follower systems, shared-control methodologies have been developed to overcome challenges such as limited bandwidth, reduced situational awareness, or the need for increased safety. These strategies combine human input with autonomous robotic behavior to optimize task execution while minimizing cognitive and physical demand on the operator.

One notable example is the Echo project and its successor, TESSA, which implement a shared-control paradigm suited to environments with constrained connectivity and minimal local expertise [31]. Originally designed to support astronauts aboard the International Space Station (ISS), the system relies on basic probe positioning by the user, while expert radiologists on Earth guide the process remotely. Robotic telemanipulation assists in maintaining precise positioning and enables safe and reliable ultrasound procedures. Though not strictly a leader–follower model, TESSA exemplifies hybrid control where responsibilities are distributed between human and machine. The technology has since been adapted to terrestrial contexts such as oil rigs, correctional facilities, and rural clinics.

Shared control is a teleoperation paradigm in which control authority is dynamically distributed between the human operator and the robot. Rather than directly executing every user command, the robot interprets operator intent and blends it with autonomous behaviors such as obstacle avoidance or trajectory optimization, to improve task performance, safety, and ease of use. This approach is particularly valuable in complex or uncertain environments, where full manual control may be inefficient or error-prone. A representative implementation is presented in [32], where the authors enhance classical artificial potential fields by introducing dynamic escape points and a soft constraint satisfaction model. Their framework enables the robot to autonomously assist the user by navigating around obstacles while maintaining adherence to the user’s intended direction.

An effective example of supervisory control is presented in [33], where the surgeon defines high-level actions, such as needle entry and exit points, using a 3D point-cloud interface, and the robot autonomously plans and executes the required trajectories. This reduces reliance on direct manipulation and improves efficiency in constrained environments. The approach is extendable to tasks like cauterizing or stapling, and includes a fast inverse kinematics algorithm to optimize arm positioning based on portal constraints. Although developed in simulation, the system is being migrated to a physical Da Vinci robot via ROS, with the aim of refining performance through real-world feedback.

Another relevant strategy in remote robot control is Programming by Demonstration (PbD), which enables robots to acquire skills directly from human demonstrations instead of being programmed manually [34]. This paradigm is particularly suitable for telemedicine, where clinicians may lack robotics expertise but possess critical domain knowledge. In [35], PbD is applied to robotic suturing through diffusion policy learning, using expert demonstrations to teach complex needle-driving motions. To ensure safety in uncertain or out-of-distribution scenarios, the system estimates epistemic uncertainty and returns control to the human operator when needed. Additionally, control barrier functions are used to enforce safety constraints throughout the execution. Experimental results in simulation highlight the robustness of this approach in handling perturbations such as dropped needles or unexpected patient motion, demonstrating that PbD can combine intuitive task specification with autonomous execution and safety assurances—qualities essential for high-stakes remote medical interventions.

### 2.2. Towards Smart End-Effector Tools for Minimally Invasive Robotic Medical Procedures

Minimally invasive robotic surgery requires precise and task-specific instruments mounted on the robot’s end-effector. Depending on the medical procedure, such as laparoscopic operations (e.g., cholecystectomy [36], or hernia repair [37]), diagnostic sampling for viral detection [3], cardiac valve repair [38], or oncology-related interventions, one or multiple robotic arms may be employed. These are typically equipped with instruments like forceps, scalpels, scissors, biopsy needles, tactile sensors [30], and various types of cameras, transforming the EE into a specialized medical tool.

Despite their functionality, most EEs operate as isolated components without connectivity to broader digital systems. This disconnect prevents the acquisition of critical data, such as tool usage metrics, force profiles, or error occurrences, hindering both quantitative performance analysis and the development of semi-autonomous or learning-enabled systems. Integrating sensing and communication capabilities at the EE level could unlock real-time monitoring, procedural feedback, and robot learning from prior interventions.

An example is presented in [39], where a 4-DoF end-effector was developed to perform remote-controlled endoscopic procedures via a graphical user interface. The system also featured a DT of a dual-arm setup to assist in otolaryngological diagnostics. However, its application is limited to clinical environments. The device’s bulkiness, weight, and dependence on cabled connections make it unsuitable for mobile or emergency scenarios such as SAR. Furthermore, the approach does not address system recalibration under changing operational contexts, an essential requirement for field-deployable telemedical robotics.

In IoRT applications, robotic systems rely on IoT end-devices to collect environmental data, enabling context-aware decision-making at the robot or network edge. These architectures are particularly relevant in remote medical interventions, where real-time responsiveness and system coordination are critical.

Leveraging IoT principles, the system described in [40] integrates a motion-tracking device as an Edge component to monitor the surgeon’s hand proximity in real time. Based on this input, virtual repulsive forces are computed to autonomously adjust the robot’s elbow position, preventing unintended collisions and improving operator safety and ergonomics—without requiring direct contact.

As we move toward 6G-enabled infrastructures, there is a growing demand for flexible service routing and data-centric architectures capable of orchestrating distributed resources across heterogeneous platforms. These paradigms prioritize efficient dataflows and low-latency performance to support mission-critical applications such as remote robotic surgery [41].

One relevant framework is the Internet of Cooperative Agents (IoCA) architecture [27], which enables integration of heterogeneous sensor networks, mobile robots, and Edge Computing nodes for field-deployable teleoperation. In this model, both human and robotic agents, or IoT sensors—and may execute computation locally, reducing the need to offload data to cloud servers.

In mobile networks, situational awareness [42,43] denotes the ability to tailor services based on an agent’s geolocation. Within IoCA, this concept extends beyond QoS to include available computational services and connectivity constraints. Network slicing and dynamic resource allocation [44,45] are employed to ensure reliable performance across agents operating under different 5G/6G slices.

To maintain situational awareness, real-time monitoring of network conditions such as throughput and round-trip time (RTT) is crucial. These metrics are linked to each agent’s Subscriber Identity Module (SIM) via mobile routers or smartphones. Positional data can be gathered from onboard sensors (GPS, LiDAR, cameras) or robot localization systems (e.g., SLAM). Additionally, multilateration techniques using WiFi, BLE, or UWB signals can provide relative positioning, particularly in SAR or indoor scenarios [46].

Depending on the use case, precise geolocation enables agents to coordinate relative to a mission reference point, such as a command center (SAR) or an ambulance (medical deployment). Agents located further from the main 5G/6G base station typically experience lower location awareness and increased latency, making cooperation between Edge nodes essential for balancing computational loads and ensuring timely data delivery.

Human agents may take on different roles based on their location awareness: deploying IoRT devices, assisting the teleoperator, or performing local supervision. Teleoperators, in particular, require the highest level of location awareness, as they demand real-time interaction with both the physical and virtual components of the system.

## 3. Teleoperation System

### 3.1. Requirements

The main challenge lies in safely making physical contact with a human using a robotic arm in non-engineered or even out-of-hospital environments. To address this, specific requirements must be met across four key areas: standardization of the EE, mobile-based teleoperation, Edge–Cloud continuum architecture, and sensory feedback delivery to the remote operator.

EE instrumentation must be lightweight, portable, and standardized to allow quick deployment and easy attachment to the robotic arm in the application scenario. The EN ISO 9409-1 standard [47] provides guidelines for the mechanical interface of industrial robots, specifically defining the dimensions and types of flanges used to connect tools or EEs to robotic manipulators. Telemedicine use cases require end-effectors compatible with a wide range of CRAs, including models such as the UR3e (3 kg payload), UR5e (5 kg), UR10e (12.5 kg), and UR16e (16 kg) from Universal Robots (Odense, Denmark); the LBR iiwa 7 R800 (7 kg) from KUKA (Augsburg, Germany); the Motoman HC10 (10 kg) from Yaskawa (Kitakyūshū, Japan); and the TM5 (7 kg) from Omron (Osaka, Japan). These robotic arms are suitable for use in operating rooms [48], as well as in outdoor SAR scenarios when mounted on large UGVs [4].

The IoRT-in-hand is a smart, detachable device designed to be mounted on the end-effector of a robotic arm. It integrates an Edge Computing unit that serves as an entry point for data from the application scenario, with onboard processing capabilities and Internet connectivity, all embedded within a compact mechanical structure. As a modular smart end-effector, the IoRT-in-hand must remain lightweight—under 3 kg—to ensure both safe and precise control by CRA and the possibility of drone delivery in outdoor SAR operations. Additionally, the device should establish a fast and reliable Internet connection as soon as it is attached and powered on.

**Figure 2:**
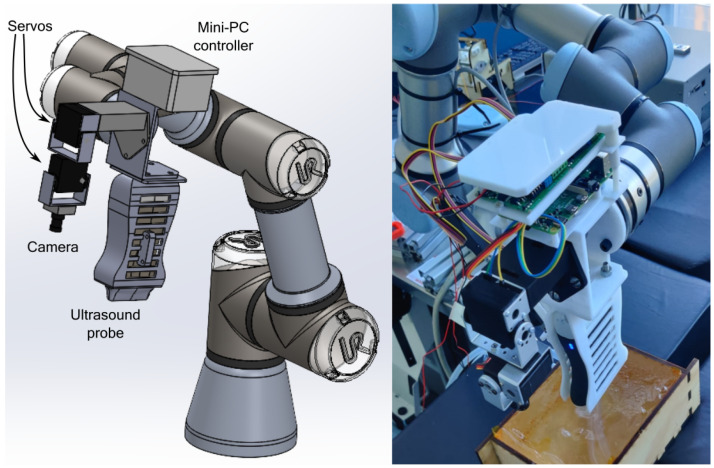
Design of the end-effector supporting the IoRT-in-hand.

Figure 2 shows the IoRT-in-hand, housed in a 3D-printed enclosure with dedicated compartments for the components required to perform tele-robotic ultrasound. These include a small RGB camera with integrated torch for subject illumination, two servos to control the camera’s pitch and yaw, an ultrasound scanner for imaging, a force–torque sensor to monitor interaction with the patient, and a mini-PC to process the data and transmit it to the remote teleoperator. The complete CAD design of the enclosure is publicly available in an actively maintained GitHub repository (https://github.com/Robotics-Mechatronics-UMA/IoRT-in-hand (accessed on 20 June 2025)), which will be updated as the prototype continues to evolve.

The teleoperation is carried out from a mobile device, such as a smartphone, without requiring additional accessories like smart glasses. This design allows any remote physician to easily control the robotic system without relying on specialized engineering tools. A key requirement is the availability of stable, low-latency communication with sufficient bandwidth to stream images to the operator in real time. For this reason, both ends of the system are expected to connect to networks with at least 5G capability, ensuring low latency, and the smooth transmission of medium-resolution images without interruptions. In this work, we evaluate the system under real-world conditions, with both endpoints operating over commercial mobile networks, and therefore, subject to variable traffic demand and performance fluctuations.

Depending on the characteristics of the application scenario, additional components or IoT end-devices may be required within the robot’s workspace to interact with the IoRT-in-hand. To support these interactions, both the visualization-potentially including augmented reality—and the control interface are integrated into a Multi-access Edge Computing (MEC) center, which is accessible from the smartphone. This setup enables the teleoperator to perceive the environment in 3D and interact with the system efficiently.

In this work, arUco markers [49] are used as connected elements in the environment, becoming part of the IoT once detected by the IoRT-in-hand. These fiducial markers are useful not only for identifying objects and estimating their relative pose within the robot’s surroundings, but also for enabling augmented reality overlays linked to the DT [50]. The innovation presented here lies in using this virtual information to provide the operator with a three-dimensional understanding of the robot’s workspace. The main challenge is rendering both the physical and virtual environments on the smartphone screen to deliver an integrated 3D perspective. To ensure low-latency operation, the system relies on a distributed ROS-based architecture deployed in the cloud. Additionally, robot and DT control commands can be issued through an MQTT broker, also hosted in the cloud. This setup enables the smartphone to remotely access and control both elements over a wide-area network (WAN) using ROS and MQTT.

### 3.2. Architecture

**Figure 3:**
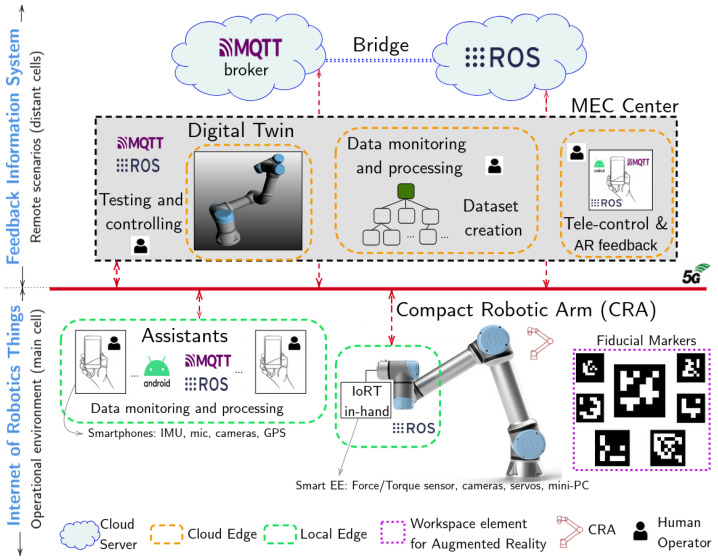
Edge–Cloud architecture for tele-robotic manipulation.

Once all IoT components are integrated into a detachable mechanism compliant with the EN ISO 9409-1-50-4-M6 standard [47], they must be interconnected with both the mobile device used for remote control and the MEC center hosting the DT server. To achieve this, we propose an Edge–Cloud architecture (see Figure 3) that enables real-time cooperation between the human operator and the robotic system, interconnected through ROS. This architecture supports the digitalization of healthcare procedures and the deployment of remote medical solutions.

Medical specialists are a limited and highly valuable resource, and it is crucial to avoid exposing them to high-risk environments such as war zones, terrorist incidents, or natural disasters. The proposed system addresses this by allowing medical procedures to be performed remotely in scenarios where expert personnel are either unavailable or cannot be physically present due to safety concerns or time constraints.

#### 3.2.1. The Feedback Information System

The following services are particularly important for supporting the teleoperator:

- Real-time visualization of the DT: This includes the display of virtual points associated with ArUCo markers, enabling the mapping of both structured objects within the robot’s workspace and specific parts of the patient’s body. To achieve this, reference markers can be placed directly on the subject such as wearable bands, so that any movement is reflected in the virtual environment.
- Access to sensory data from the IoRT-in-hand: The operator can view real-time RGB camera feeds and monitor the forces exerted by the end-effector.
- Control of end-effector motion: The system allows the teleoperator to command Cartesian velocities to the end-effector remotely.
- Real-time ultrasound visualization: Live ultrasound imaging is streamed to the operator to support remote diagnosis during tele-robotic procedures.
- Safety pose management: At any time, the robot can be commanded to return to predefined safety poses. These trajectories can be previewed in the virtual environment, as the app developed enables DT control via MQTT commands.

All computing tasks involved in the system are executed remotely, outside the application scenario. The proposed architecture distributes these tasks across multiple hosts using a fog computing approach, allowing the IoRT-in-hand to be managed and controlled without requiring local configuration. The device is designed to operate with a predefined setup, enabling autonomous functionality once deployed. Core middleware components, such as the ROS communication framework and the MQTT broker, are hosted in the cloud, independent of their physical location. Meanwhile, the DT server is located in an MEC center, which also manages the overall network infrastructure. All connections are established through a VPN coordinated from the MEC center. System-level operations, such as testing, networking, control, and monitoring, are integrated into a Fog Integration System (FIS). The smartphone used to control the robot acts as a fog device within the MEC layer, even if its physical location and Internet provider differ from those of the DT server.

The FIS encompasses all mission-critical tasks performed on dedicated computing nodes located in remote network cells, including data monitoring, processing, remote control of the CRA, and dataset generation. Within this framework, the MEC center hosts a DT server capable of emulating the CRA’s movements in a virtual environment, created in VEROSIM. This allows synchronization between the real robot and its virtual counterpart, regardless of whether the control is autonomous or teleoperated. As a result, the remote operator can observe the CRA’s actions in real time through the DT server, which streams the virtual scene to the operator’s interface.

#### 3.2.2. The Internet of Robotic Things

Within the main mobile cell, where the CRA has to perform the medical application, there are two classes of Edge devices: the IoRT-in-hand, and the smartphones carried by the medical assistants. Thus, smartphones can have different roles within the IoRT or the FIS, depending on whether they act as information providers or remote processors and task monitors, respectively. An Android app, based on the ROS-Mobile app [51], has been developed (https://github.com/Robotics-Mechatronics-UMA/Adhoc-ROS-Mobile (accessed on 20 June 2025)), to integrate the capabilities of MQTT clients and ROS nodes on a smartphone. Thus, the smartphone screen serves as a user interface for communication between the CRA and the DT. The screen displays both the images from the camera in the smart EE and the DT server, simultaneously providing two perspectives to the teleoperator. Specifically, the bridge in the cloud allows the teleoperator to synchronize and control, in real-time, either the DT or the CRA from the app. Thus, it is possible to move the DT without seeing the physical robot, which allows the teleoperator to improve their skills. Moreover, any assistant using their smartphone can access to the images from the virtual environment, specifically, the DT, and the onboard RGB camera (eye-in-hand).

The IoRT includes sensor networks distributed between the CRA, the assistants and their workspaces, such as cameras, microphones, physiological and industrial sensors, GPS, as well as other kinds of elements to include augmented reality capabilities. Fiducial markers, such as, ArUCo markers are typically used to estimate the distances between the end-effector and the objects within a structured environment. Moreover, we use them to load extra virtual points around the DT, hosted on the MEC center.

All the devices distributed between the Edge and the Cloud have processing and storage capabilities, and are connected to the CRA controller and to the IoRT-in-hand through a VPN, being able to exchange information between the application scenario and the FIS [52]. The most important information from the application scenario is related to the CRA parameters, such as joint values, end-effector position, force and torque intensities on the tip, processed images from the cameras, and transformation matrices.

#### 3.2.3. Interoperability Between the CRA, IoRT-in-Hand, Android App, and DT Server

To ensure the safe movement of the CRA within the human subject’s environment, the communication system must provide low latency and high bandwidth—requirements that can be met by a 5G network. For this reason, a 5G connection is recommended to link distant Edge and Fog devices. The core challenge is to remotely control the robot to physically interact with the human using the IoRT-in-hand device, which demands smooth and delay-free operation. In this context, minimizing latency in the video stream from both onboard cameras is essential to provide responsive visual feedback during teleoperation. To achieve this, efficient image compression codecs should be applied prior to transmission, or streaming should be limited to situations where it is strictly necessary.

However, the captured RGB images do not need to leave the IoRT-in-hand device, since the detection of ArUco markers—used to locate objects within the robot’s workspace—is performed locally. This edge-side processing ensures minimal display latency, as the pose estimation of the referenced objects is carried out directly at the IoRT-in-hand. The resulting data is then transmitted in real time via MQTT to the DT server, allowing the teleoperator to visualize virtual points corresponding to those objects within the shared digital workspace. Data such as position, orientation, and transformation matrices are computed on the Edge device and transmitted to the DT server using MQTT. These matrices are computed locally using ArUco marker detection via OpenCV. The detected rotation and translation vectors are converted into 4 × 4 homogeneous matrices, which are then chained with pre-calibrated transformations to map the detected marker pose into the robot base frame. The pose of the end-effector, obtained via an ROS topic, is also incorporated into the final transformation. To improve robustness, a temporal median filter is applied over a sliding window before publishing the resulting matrix via MQTT to the DT server.

In addition, the MQTT Extension for VEROSIM [53] enables the external client integrated into the teleoperator’s smartphone to interact with the DT, while access permissions can be configured to restrict other external clients from modifying property values. This MQTT plugin also facilitates communication between the virtual environment and the physical world by detecting ArUCo markers. Both the physical robot and the DT can be controlled via the Android application, which integrates external MQTT clients and ROS nodes through an ROS-MQTT bridge [54], leveraging both timer-based and event-based publishers, as well as subscribers. In this way, a mobile device such as a smartphone can control both the DT and the CRA.

To address interoperability across heterogeneous communication standards, a cloud-hosted ROS-MQTT bridge was implemented. This bridge enables forwarding messages from ROS subscriber nodes to MQTT topics, which are published by MQTT clients connected to a broker — in our setup, a public Mosquitto broker deployed in the cloud. This approach was selected specifically to overcome the limitations imposed by Network Address Translation (NAT), which often impedes bidirectional ROS communication across networks. By centralizing the message flow through a cloud-based MQTT broker, ROS data can be exposed securely to any external client, bypassing the need for port forwarding or static IP configuration.

Once ROS messages are forwarded via the bridge, MQTT clients running on the onboard PC (attached to the IoRT-in-hand) publish the data externally over the WAN. Any authorized external MQTT client can then receive the data by subscribing to the appropriate topics (e.g., using a command such as mosquitto_sub -t /topic_from_ros -h [MQTT_BROKER_IP]). This mechanism also allows the DT, hosted on a remote Verosim server, to subscribe to real-time data streams using the aforementioned MQTT plugin, ensuring seamless synchronization between the physical system and its virtual counterpart. Thus, all components in the system communicate asynchronously via their native pub/sub paradigms—ROS or MQTT—without requiring full protocol conversion.

The custom Android application developed for this system includes both MQTT clients and native ROS nodes, enabling it to selectively publish and subscribe through either protocol. For example, the app may publish physiological sensor data to the DT via MQTT, while simultaneously sending motion commands to the robot via ROS. Importantly, this architecture avoids unnecessary bridging of all external ROS topics to MQTT, allowing selective and efficient data exchange. It also ensures compatibility with both ROS 1 and ROS 2 environments.

In the ROS 1 case, this design eliminates the need for complex ROS-TCP configurations that would otherwise require opening a wide range of ephemeral ports. For ROS 2 deployments, we employed Cyclone DDS as the middleware, configuring all nodes with the same ROS_DOMAIN_ID (ID 0)—the default and immutable value on Android platforms unless rooted (which was not desirable in our case). Further architectural details and implementation clarifications have been added in this revised version of the manuscript.

The ROS communication infrastructure and the MQTT broker are hosted in the cloud, and all components are interconnected via a Virtual Private Network (VPN) based on ZeroTier, which eliminates the need for Network Address Translation (NAT). Thus, every host is assigned an IP address within the same virtual network, being accessible through ROS [55] and MQTT. In addition, the architecture requires the routers’ ports for cameras and DT application to be open.

**Figure 4:**
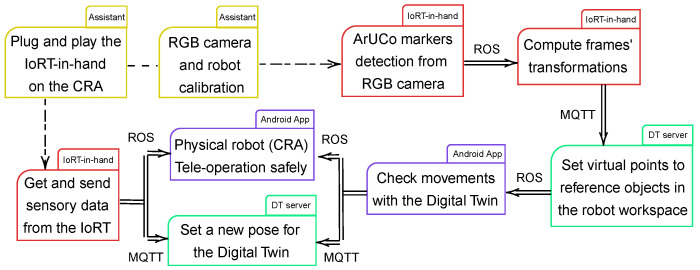
System interoperability for remote teleoperation.

Figure 4 summarizes the followed interoperability scheme to run the teleoperation system. First, a medical assistant puts the IoRT-in-hand on the robot EE and switches it on. After that, they calibrate the RGB camera and robot [56] if necessary. Usually, it is only required for the first use of a specific workspace.

Then, the IoRT-in-hand gathers information about the robot’s interaction with its surroundings, including RGB and ultrasound images, forces at the end-effector, and ArUco marker detections. Once fiducial markers are detected within the robot’s workspace, their spatial poses are estimated in real time by composing a chain of transformations: from the robot base to the EE, then to the camera, and finally to each detected marker. These transformation matrices define the coordinate frame of each object and are transmitted to the DT server (running VEROSIM), where they are visualized as virtual points anchored to their corresponding frames.

Some of the markers are statically placed to reference fixed elements in the workspace, while others are attached to the human subject using elastic bands. These dynamic markers are identified by their known IDs and enable real-time tracking of the subject’s limbs. Finally, the DT enables validation of the spatial configuration and planned motions before safely teleoperating the CRA.

## 4. Robotized Telemedicine Implementation

For illustration and validation, we present a case study in which a robotic manipulator is controlled in real time to perform a remote ultrasound scan. Next, the three major parts of the architecture are described below: the application scenario within the main cell where the CRA is; a remote cell, where the smartphone used for teleoperation is considered a Fog-Edge device; and the FIS, where several fog devices coexist by exchanging information with the application scenario and Cloud.

### 4.1. Application Scenario Setup

First, it is necessary to prepare the subject on a stretcher, and perform the eye-in-hand (RGB camera) and robot calibrations. In principle, this only needs to be performed the first time the IoRT-in-hand is placed on the EE of the CRA, unless environmental conditions change drastically, and recalibration of the camera is necessary to ensure the accuracy of the estimates. Thus, camera calibration is needed to obtain its parameters, and then be able to apply AR, through the ArUCo markers.

For camera calibration, the implemented methodology is based on chessboard calibration images, where it is necessary to obtain all the corner points inside the chessboard, discarding the outer ones. The process involves capturing, at least, 10 images of the entire chessboard, taken them in different planes to avoid degenerate case, from different angles and positions, detecting the corners of the images, and then using optimization algorithms to find the camera parameter values that best fit the observations. Then, for every corner point, a relationship between image and world points is achieved. The mentioned parameters allow the camera to map real-world 3D points to 2D points in the image.

**Figure 5:**
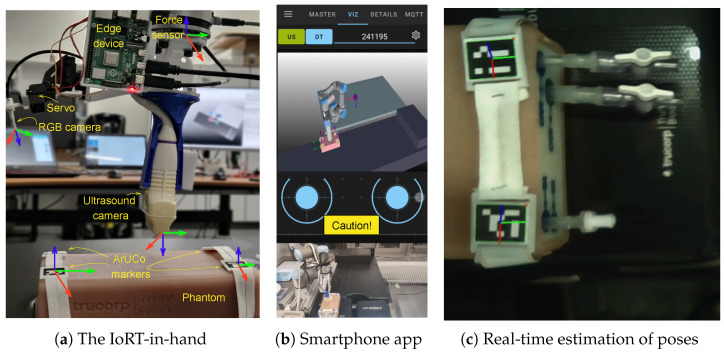
Testing setup: The tele-robotic echography is tested on a phantom, being the DT synchronized with the CRA. The transformation matrices are calculated on the Edge device, and poses estimated are sent via MQTT to the DT server to display the virtual target points for the CRA.

For guiding the robot to the target body surface, two straps with ArUCo markers are adjusted to the subject (see Figure 5a, where a phantom is used for testing). The rest of the workspace is mapped using a tip on the end-effector for the robot calibration. The virtual environment has been emulated by referencing objects in the robot workspace, such as the bed, the robot table and the relative distances to the objects. Every detail has been taken into account while creating the virtual model and DT in VEROSIM.

Once the IoRT-in-hand is switched on, it is connected to a wireless network whose credentials are saved in the Edge-ç device configuration. After that, the ROS package is launched automatically, since it is registered in the bash file. For this prototype, a mobile network is the Internet source of the whole testing setup.

Regarding the rest of the IoRT, it includes the cameras worn by the medical assistants, being the video stream available in the smartphone of the teleoperator via ROS.

Each time an ArUCo marker is detected, its pose is sent to VEROSIM, via MQTT, in order to establish a virtual point with the position and orientation referred to the object, with respect to the camera’s reference system. In this way, the distances between the body to be scanned and the ultrasound camera are estimated. Moreover, the feedback provided by the force sensor gives the teleoperator an idea of whether or not he is touching the subject.

The chosen CRA is the UR3 manipulator (Universal Robot, Odense, Denmark). The work envelope is 500 mm, and the reach of all its joints is ±360º, except for the last one, which has infinite rotation. Its three wrists have a speed of 360º/s, while the first three joints have a speed of 180º/s. Representative speed values for robotic medical interventions are in the range 2–170 mm/s [57,58]. However, for remote operations, the maximum speed of 30 mm/s has been set for safety reasons. The CRA controller is based on the real-time data exchange library (https://sdurobotics.gitlab.io/ur_rtde/ (accessed on 20 June 2025)). The relative position of the CRA to the bed requires prior calibration. Additionally, this allows the virtual environment to be preconceived and fully structured in the DT environment. At the robot’s end, an Edge device is designed to include a 6-DOF force sensor (OptoForce, Budapest, Hungary), a local edge, a camera servo stage, and an ultrasound probe. The local edge is implemented using a Raspberry Pi 4B. The RGB images captured by the camera are processed by the local Edge to detect the ArUCo markers, which enables the determination of the relative position between the CRA and the patient. The servo stage allows the camera to be controlled, changing the viewing angles. The edge device also acquires ultrasound imaging and measures applied force. The vertical force is continuously monitored, and a warning message is sent via MQTT if it exceeds a threshold value.

In addition to the applied force, the relative position is also streaming to the FIS via MQTT in real time, thus being able to estimate and visualize the position of the ArUCo markers both in the RGB image and in the virtual environment (see Figure 5). Meanwhile, data generated by the physiological sensors on the patient are transmitted via MQTT if they are lightweight enough to be sent efficiently. Here, we use a wearable device based on a photoplethysmography (PPG) sensor and an ESP32 board to monitor heart rate and transmit this data in real time.

Regarding the dataflow exchanged through subscriber/publisher ROS nodes, there are the images captured by cameras worn by the medical assistants, the values of the robot’s joints and pose messages, as well as the warnings sent to the teleoperator based on the readings of the force sensor located at the tip of the robotic arm. In addition, the virtual joysticks (on the smartphone screen) with which the teleoperator interacts send the speed commands to the robot controller to move it in the Cartesian coordinate system.

The application scenario (see Figure 5) consists of a bed on which the patient would lie down. The local reference system of the ultrasound camera has been drawn in Figure 5a, being the Y-Axis parallel to the bed. A table on which the CRA is placed is close to the bed. The region of interest for ultrasound scanning is defined by two straps with ArUCo markers and is covered by the robot workspace.

Finally, the TCP/IP protocol is used to transmit images taken by the RGB and ultrasound cameras, and the virtual environment where the DT is hosted. The necessary ports are enabled on the routers of each network end.

### 4.2. Remote Cell

A human agent, usually an MD, teleoperates the CRA from a remote cell using a smartphone. For this purpose, the ad hoc ROS-Mobile is designed with two displays. The user can select to view video streaming hosted on the web, including IP camera streams and ROS images from other smartphones [55]. The top display can show either the ultrasound imaging or the DT scenario, determined by the two buttons on the left upper corner (see Figure 5b). The bottom one displays real-time ROS images from any RGB camera, such as those on assistants’ smartphones. By this means, we aim to facilitate the control of CRA for the teleoperator. In addition, the app allows the user to control the CRA movement through two virtual joysticks. A dead man’s switch is enabled for both joysticks to automatically return to zero when the user stops touching the mobile screen.

Moreover, a new ROS subscriber node is created to warn the user about the pressing forces that the EE exerts on the patient. The pressing force is categorized into three levels and represented by different colors (green for safe, yellow for caution, and red for dangerous) to signify the potential discomfort that the patient may experience.

The local Edge device onboard the CRA hosts an ROS publisher node which sends a value of type UInt8 (0, 1, or 2) depending on the measured forces on the ultrasound probe. Two limits are established according to the patient’s pain. The color risk (green, yellow, and red) associated with the measured pressure on the tip is shown on the smartphone screen thanks to the new ROS subscriber (see Figure 5b) running in the ad hoc ROS-Mobile app. In addition, the app includes a new tab associated with MQTT clients hosted on the user’s smartphone to allow information exchange with other MQTT clients in the tele-robotic system.

### 4.3. Digital Twins and Augmented Reality for the FIS

The FIS consists of three MEC centers: a health center, a data center, and a server running the DT.

In the health center, the human agent is typically an MD whose role is to supervise the teleoperation and provide diagnosis suggestions by watching the stream-back ultrasound imaging and the patient’s physiological signals.

The data center collects and saves the data from both the remote cell and the application scenario. Specifically, data generated during the experiments are saved in a rosbag. MQTT topics and images transmitted via TCP/IP (https://motion-project.github.io/ (accessed on 20 May 2025)) are converted to ROS topics before being saved.

The DT runs in VEROSIM, a virtual testbed implementation for 3D robot simulations [59]. It is constructed in a Cloud/Edge server where an human agent (typically an engineer) is responsible for supervising the procedure. The DT receives MQTT messages from the remote cell for synchronizing the posture between the CRA and the DT. By this means, the DT enables the human agent in the remote cell to assign multiple waypoints and conduct a dry run in the virtual environment. Also, an interface based on Deskreen (https://github.com/pavlobu/deskreen (accessed on 20 May 2025)) is designed for capturing the DT display and streaming it via TCP/IP. This allows the human agent in the remote cell to visualize and gain more insights into the CRA movement from the virtual environment in a different perspective.

## 5. Experiments and Results

Two types of telemedicine exercise experiments were conducted for the medical implementation of the proposed tele-robotic system. The first type was designed to characterize the system based on a LAN, where multiple local teleoperations (LTs) were carried out to test the different parts of the system. In the second type, a series of remote teleoperations (RTs) were conducted between two laboratories located in Malaga (Spain) and Odense (Denmark), with a VPN established between the two endpoints of the network. The end in Malaga used a 5G network, while the end in Odense used a 4G-level network. Figure 5 illustrates the application scenario for both types of experiments, where the teleoperator was the same person. The aim of these experiments was to measure five key parameters to evaluate the feasibility of using our IoRT-in-hand in practical applications, including forces on the end-effector, the number of warnings given to the teleoperator, time to do the telemedicine, time in contact with the body, and latency between the network ends.

### 5.1. Evaluation Metrics

A well-designed teleoperation architecture should provide an intuitive user experience, reducing the occurrence of operational errors. Taking the complex anatomical geometry into account, controlling teleoperation remotely can be challenging for operators. The efficacy of a teleoperation can be measured by the ability to quickly locate the target without exerting excessive force on the patient’s body during the scan, which can be quantified by the percentage of time spent performing the scan (τ) compared to the total time ($t_{t}$).

(1) $$\psi =\tau /t_{t}$$

In addition, we define a penalty term Ω to include a weighted relevance on the number of warnings triggered due to the exceeded pressing force on the body during tele-robotic echography. Two warning thresholds are pre-set to indicate the maximum allowable force exerted without causing the patient discomfort (yellow alert) or pain (red alert). Specifically, Ω is defined as follows: a red warning counts four times higher risk than a yellow warning.

(2) $$\Omega =4\times \omega_{R}+\omega_{Y}$$

The final performance index is defined as φ:

(3) φ=α×ψ−β×Ω/(1−ψ)

where α and β are the weights for balancing the operational efficacy and the penalty due to excessive pressure. After some practical considerations, the values of α and β are chosen to be 230 and 0.25, respectively. This is not intended as a precise mathematical expression but as a way of measuring the quality of teleoperations in percentage: φ∈(0,100)[%]. It is assumed that an MD performing a scan manually should achieve a 100%.

### 5.2. Experiment 1: Characterization of a Local Implemented System

The aim of Experiment 1 was to test the tele-robotic system within the same LAN network, except for a dedicated computer representing the cloud and the smartphone used for LTs, which operated over a 4G network. The goal is to characterize the overall performance of the system (using the φ index) when operating within the same LAN network. The agents and MEC centers were connected to a WiFi-enabled router, and the LAN was linked via a Virtual Private Network (VPN) to both the smartphone and the cloud, where the ROS-based communication infrastructure and the MQTT broker were deployed. The visualization of the DT, as well as the camera streams, functioned smoothly, and the teleoperator was able to perform the LTs from a smartphone using its own mobile data connection. The app retrieved all necessary data through the VPN. With this setup, the maximum end-effector velocity associated with full joystick deflection was set to 30 mm/s to ensure safe operation of the robotic agent.

Table 1 presents the results from four consecutive LT runs (LT1, LT2, LT3 and LT4). There was an improvement in the φ as the teleoperator gained experience with the app. LT1 and LT2 had a similar duration and φ. Subsequently, the same human agent performed LT3 and LT4, reducing the $t_{t}$ and significantly improving the φ, which was close to 100%. After these runs, the teleoperator felt confident working at speeds even higher than the operation limits set in the joystick app configuration.

**Figure 6:**
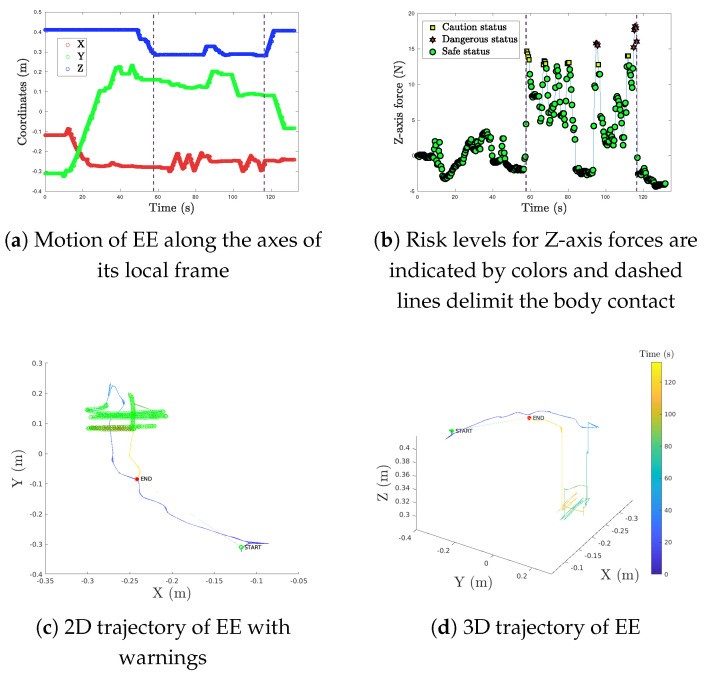
Results for LT3 (φ = 62.07%).

Figure 6 shows the EE’s trajectories for the CRA during LT3, to illustrate what kind of movements are performed by the system during Experiment 1. Before scan time, the EE lost height (in blue) as it approached the phantom. During the scan time, two zig-zag sweeps were performed (see Figure 6a), as seen in the X-axis movement (in red). At the beginning and the end of these two scans (i.e., when contact started), some over-pressure occurred (see Figure 6b), which triggered warnings on the smartphone’s screen, as indicated in Figure 6c. The start point in Figure 6c,d represents the start of the CRA’s movement, while the endpoint indicates a safe landmark for the patient, being chosen by the teleoperator as a good moment in the return trajectory to stop the CRA.

### 5.3. Experiment 2: Remote Teleoperation on a Volunteer

**Figure 7:**
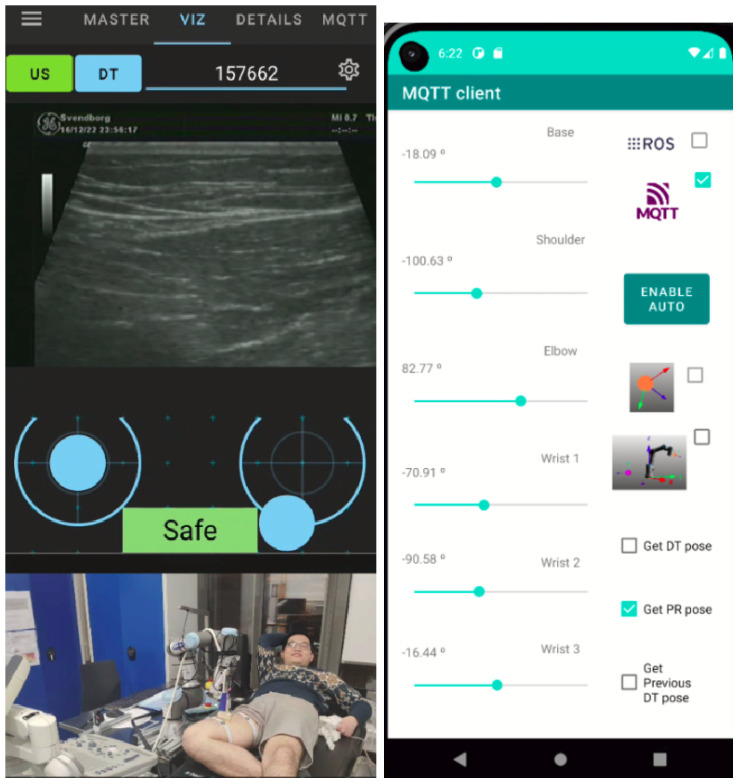
RT for robotic ultrasound scan on the subject’s leg using the ad hoc ROS-Mobile. The integrated MQTT client synchronizes the movements of both DT and RA.

The second type of experiment was conducted between two distant locations to evaluate the tele-robotic system performance. In these experiments, the teleoperator and the cloud were in Malaga, while the CRA, the DT, the subject and the assistants were in Odense, 2300 km away. A series of RTs were conducted aiming to find the femoral artery on the subject’s thigh (see Figure 7). For RTs, the maximum EE velocity was limited to 8 mm/s because higher velocities gave rise to mistrust on the part of the teleoperator. The velocity commands from the smartphone were always given at 60 Hz. However, the CRA’s behavior was quite accurate up to 20 mm/s, at which point the ROS buffer tended to saturate, translating into discontinuous movement.

Table 2 presents the results from three consecutive RT runs (RT1, RT2, and RT3) carried out on the same day by the same teleoperator as in LTs. On previous days, up to fourteen remote tests were performed for operator training and developing data logging in ROS bags. The table indicates that the teleoperator had previous experience with the app interface and made fewer errors as the exercise was repeated. The noticeable decrease in φ in subsequent runs was due to the need for verbal interactions between the teleoperator and the assistant to record the dataset synchronously during RTs. In addition, each new RT run generated stress on both human agents, directly influencing φ.

**Figure 8:**
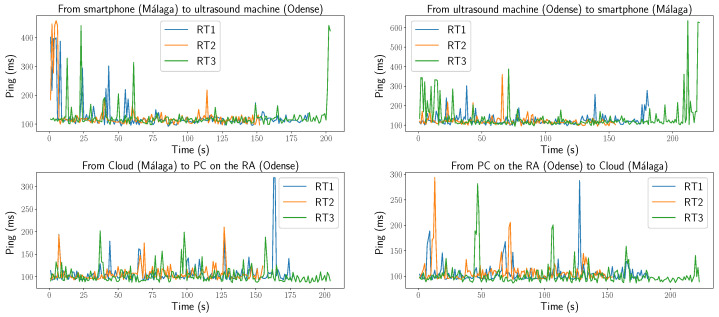
Ping values between the teleoperator and the application scenario during RT1, RT2 and RT3. The mean value was 116 ms, the distance being 2300 km.

Figure 8 shows the latency between devices at each end of the network. The implemented code for controlling both the CRA and the DT, as well as retrieving information from them, can be executed at either network endpoint. However, both processing and latency performance improved when the code was executed near the CRA. Latency was estimated as half of the round-trip time (RTT) measured using ICMP ping. The maximum latency peak recorded was 318 ms (i.e., a round-trip time of 636 ms), while the minimum was 43 ms (ping = 86 ms). The average latency during the RTs was 58 ms.

## 6. Discussion and Lessons Learnt

The proposed metrics offer an intuitive framework for quantifying teleoperation performance by integrating operational efficiency with safety considerations. However, the weighting scheme, particularly, the decision to penalize red alerts four times more than yellow alerts, was derived from preliminary testing and practical insights. While this reflects the greater clinical relevance of excessive force warnings, the approach lacks formal clinical validation. Similarly, the coefficients used in the global performance index (α=230 and β=0.25) were selected empirically to balance time efficiency and safety, but their optimality and clinical significance remain unverified.

The use of both MQTT clients and ROS nodes to control our IoRT-in-hand system was driven primarily by integration constraints and modularity considerations. ROS is a standard middleware in robotics research due to its powerful capabilities for real-time data streaming, robot control, and ecosystem of tools. However, one of our core software tools, VEROSIM, did not have native support for direct ROS integration or ROS-based data extraction. Instead, VEROSIM provided built-in mechanisms compatible with MQTT, an industry-standard lightweight protocol widely used in IoT applications for reliable, low-bandwidth messaging.

Rather than developing a complex ROS integration plugin from scratch for VEROSIM, the decision to use MQTT was taken strategically to simplify and accelerate the integration process. MQTT acted as a reliable and straightforward communication bridge, transmitting critical simulation and monitoring data from VEROSIM to our ROS-based system components. This approach reduced development complexity, promoted modularity, and allowed independent testing and debugging of the simulation environment (VEROSIM) and robotic control software (ROS). Additionally, MQTT provided benefits such as message persistence, simple payload structures, robustness to unstable network conditions, and suitability for cloud-based monitoring systems, complementing ROS functionalities effectively.

Although this design introduces a certain level of heterogeneity by combining two communication protocols, the clear interface between ROS and MQTT components resulted in a flexible and practical solution that proved robust in our experimental scenarios. Future work may explore alternative integration solutions, such as direct ROS plugins for VEROSIM or standardized middleware like ROSbridge, to further streamline the architecture.

Furthermore, the use of an Android application capable of simultaneously integrating MQTT clients and ROS nodes facilitated comprehensive testing of the interaction between the simulation environment (VEROSIM) and ROS-based robotic components. This hybrid design enabled remote operators to interact intuitively with the DT via MQTT without the necessity of direct access to the ROS network, providing an extra security layer by isolating the physical robotic equipment from external connections. Specifically, commands sent remotely via MQTT were initially executed only on the DT, with actual movements on the physical robot possible exclusively through ROS-based communication. Thus, physical robot interaction was protected by requiring explicit acceptance into the ROS network and confirmation by a human operator physically present at the robot’s location. In this way, the combined MQTT-ROS architecture enhanced accessibility and usability for remote testing, while ensuring operational safety for physical equipment.

Moreover, the modular design of the system opens the door to further extensions aimed at improving runtime coordination and observability. For instance, exposing dedicated ROS services to signal the start of robot motion or to manage teleoperation session boundaries could support synchronized logging across distributed components, as well as enable explicit operator authorization mechanisms. While such features were not included in the current implementation, their integration would align naturally with the existing architecture and enhance both temporal traceability and operational safety.

Studies have shown that latency begins to affect teleoperator perception from as low as 10–20 ms [60]. As it increases, its impact becomes more pronounced. For this reason, operator training plays a critical role, as it can help mitigate the delays introduced by each component of the system—such as the DT server, ROS network, MQTT broker, cellular communication links, or onboard PC processing. Among these, communication latency typically represents the most limiting factor. Nevertheless, once overall latency approaches 300–320 ms, the operator’s ability to maintain accurate control deteriorates significantly, with fine manipulation and tracking performance becoming severely compromised.

Despite these constraints, the operational performance of the system proved resilient to the combined effects of delay throughout the teleoperation loop. These include network latency between the smartphone and remote servers (in our case, average RTT across trials was approximately 58 ms), touchscreen input latency (70 ms [14]), and processing delays associated with onboard computing at the robot’s end-effector. Visual processing and reaction time of the operator also contribute substantially to total delay, with typical values reported between 150 and 300 ms [61]. Notably, while modern smartphones feature display refresh rates of 120 Hz or higher (i.e., 8–9 ms per frame) and touch sampling at 240 Hz (4 ms intervals), these values are significantly lower than human perceptual and motor reaction times. Therefore, the primary bottleneck in closed-loop control is not the device hardware, but human visual-cognitive latency.

The operator was located in a 5G NSA coverage area, and three consecutive RTs were conducted. The average RTT measured in these trials was below 60 ms. Despite the accumulation of delays from communication, perception, input, processing, and actuation, the total response time between command input and observed robot motion remained within acceptable bounds for stable, real-time operation. The use of predictive visualization via the DT further mitigated the perceptual effects of latency, allowing the operator to preview actions before confirming them.

In addition, the Android application developed for this system supports the execution of autonomous motions between two points in space. These virtual target positions can be defined within the Verosim environment and subsequently used to command the robotic manipulator to move autonomously toward a desired initial pose. This functionality enables the operator to pre-position the end-effector at a convenient starting point before initiating manual teleoperation, thereby improving overall task efficiency and reducing operator workload.

Finally, although formal control-theoretic stability analysis (e.g., Lyapunov or frequency-domain methods) was beyond the scope of this work, empirical results indicate that the combination of moderate latency, system modularity, and human-in-the-loop validation led to reliable teleoperation performance. Future improvements may focus on closed-loop synchronization and delay compensation to further enhance operation under bandwidth-constrained or high-latency conditions.

### Comparison with Existing Approaches

Previous research has successfully utilized DT platforms to validate robotic surgery training trajectories within controlled environments. For example, the system presented in [62] simulated truncated cone- and pyramid-shaped trajectories in a virtual environment to evaluate the feasibility of Remote Center of Motion (RCM) control prior to execution. Their work focused on laparoscopic surgery training, emphasizing local setup, haptic precision, and offline validation. In contrast, our system targets remote ultrasound-based procedures, enabling real-time DT feedback and distributed operation. This architecture lays the groundwork for future implementations of more advanced interventions, such as REBOA (Resuscitative Endovascular Balloon Occlusion of the Aorta). While their setup enables both on-site and off-site teleoperation and integrates multiple cameras and haptic interfaces, the entire configuration is physically centralized in a training room with all components locally connected. In contrast, our system focuses on remote deployment scenarios where a medical specialist may not be physically present, emphasizing distributed interoperability over long distances. In addition, we use VEROSIM instead of RoboDK, since VEROSIM is specifically designed for real-time simulation of cyber–physical systems and supports continuous interaction with external data sources, such as ROS or MQTT, enabling dynamic visualization of environments that change during operation. In contrast, RoboDK is primarily intended for offline programming and trajectory validation in industrial settings, focusing on predefined robot motions and lacking native support for real-time updates or distributed system architectures. Unlike centralized setups, our architecture decouples the DT server from the operating room, enabling interaction and visualization from remote locations without compromising responsiveness or spatial coherence. Rather than learning from predefined virtual trajectories, our system processes real-time visual and force data locally on the IoRT device and transmits transformation matrices to the DT for visualization and validation. This approach enables dynamic, live spatial awareness—particularly valuable when tracking human body parts through elastic-mounted fiducial markers—and facilitates remote robotic interventions beyond laboratory conditions. Furthermore, our distributed architecture with integrated MQTT enables data extraction from any MQTT client with access permissions (e.g., via VPN). This eliminates the need to be within the robot’s ROS network to access real-time data. Additionally, the DT can be publicly monitored, as it is streamed directly from the DT server. This functionality allows external professionals to observe procedures remotely and provide expert feedback—enhancing educational outreach and collaborative diagnosis.

Our IoRT-in-hand device, mounted at the robot’s wrist, integrates multiple sensing modalities—including a force–torque sensor, an RGB camera, and an ultrasound imaging module—alongside a mini-PC with real-time processing capabilities and built-in internet connectivity. This compact and self-contained design enables local computation of spatial transformations and intelligent processing of visual and haptic data directly at the point of interaction. The resulting transformation matrices and relevant features are transmitted to the remote DT server for synchronized visualization and decision-making.

Unlike the design in [63], which focuses primarily on mechanical design and user interface for non-expert teleoperation, and includes only basic perception and interface-level feedback, our system supports closed-loop, distributed operation with real-time spatial awareness. While their work briefly mentions obstacle avoidance as part of the user assistance features, it is not technically detailed. Such a feature could be incorporated into our system in the future to assist with path planning; however, we consider impedance control strategies to be more critical in medical contexts that require safe physical interaction with the human body.

In addition, this system [63] leverages NVIDIA Isaac Sim as a DT platform, allowing realistic simulation, visual feedback, and motion planning in dynamic environments. Its support for algorithms like Riemannian Motion Policies (RMPs) and the recent addition of a Graphical User Interface (GUI) make it well-suited for developing and tuning teleoperation strategies, especially during the design phase.

The IMMERTWIN project [64] builds upon concepts introduced by TELESIM, implementing a VR-based DT platform that relies on real-time 3D point-cloud streaming from ZED2i stereo cameras for enhanced teleoperation and spatial understanding. Their setup was deployed locally on a high-performance workstation (Intel i7-10700F and NVIDIA RTX 3060 Ti), located in the same room as the robotic system. Despite this, they faced performance bottlenecks and had to limit image resolution to 720p and streaming rates to 10 Hz to maintain responsiveness. The IMMERTWIN project builds upon concepts introduced by TELESIM, implementing a VR-based DT platform that relies on real-time 3D point-cloud streaming from ZED2i stereo cameras for enhanced teleoperation and spatial understanding. Their setup was deployed locally on a high-performance workstation (Intel i7-10700F and NVIDIA RTX 3060 Ti), located in the same room as the robotic system. Despite this, they faced performance bottlenecks and had to limit image resolution to 720p and streaming rates to 10 Hz to maintain responsiveness. In contrast, our IoRT-in-hand architecture promotes distributed processing, with computation and compression performed directly on the sensing unit mounted at the end-effector. This unit includes a Raspberry Pi 5, whose enhanced processing capabilities proved sufficient to detect ArUco markers and compute transformation matrices in real time. The processed data is then transmitted using advanced image compression codecs and lightweight protocols such as MQTT, enabling high-resolution, real-time communication without requiring the DT server or operator to be co-located with the robot. This design supports greater flexibility and scalability, particularly for remote medical intervention scenarios.

Despite the current advancements in distributed processing, the DDS standard in ROS 2, the processing power and compactness of modern computing units, the sampling rates of sensors, the resolution of onboard cameras, and the ability to integrate all of this with DTs, to the best of our knowledge, our architecture is the first to integrate the visualization and control of a DT from a remote smartphone. This capability enables lightweight, mobile teleoperation and monitoring, extending access to critical system feedback and control beyond desktop-based setups, and marking a step forward in the democratization of remote robotic intervention.

## 7. Conclusions and Future Work

A novel Cloud–Edge architecture based on the IoCA framework [27] has been introduced, specifically designed for robotic manipulators in telemedicine applications. This architecture enables the execution of medical procedures in scenarios where specialized physicians are not available, by supporting intelligent, location-aware interactions between agents—robotic or human, according to their identity and physical context. The integration of MQTT clients and ROS nodes ensures seamless data exchange and interconnection among distributed digital components. Agent collaboration is reinforced through the application of IoRT principles and the incorporation of a DT, enabling safe and coordinated control of both the DT and the CRA, either independently or simultaneously. This configuration allows the teleoperator to receive real-time visual feedback, improving perception and operational precision. Additionally, the application integrates IoRT and DT information to provide comprehensive visual input, enhancing the reliability and effectiveness of remote medical interventions.

The system was validated in a 2300 km tele-ultrasound trial between Spain and Denmark, using a 6-DOF robot equipped with the IoRT-in-hand device. The operator used a 5G-connected smartphone to visualize the DT, monitor force feedback, and teleoperate the robot in real time. Average round-trip latency remained below 60 ms, confirming the system’s reliability under real-world network conditions.

The full system architecture included five distributed components:

1. A smartphone in Malaga, used for DT and camera visualization, as well as teleoperation;
2. A PC in Malaga, used for monitoring and data recording;
3. The robot controller PC in Odense;
4. A cloud server hosting the DT (VEROSIM) and the MQTT broker;
5. The onboard PC integrated into the smart end-effector (IoRT-in-hand), responsible for ArUco marker processing and MQTT communication through NAT.

These experiments confirmed the technical viability of the IoRT-in-hand concept, which enables remote medical specialists to extend their physical interaction capabilities using a lightweight, smart end-effector that integrates sensing, control, and communication at the edge. The combination of real-time DT feedback, Edge–Cloud coordination, and mobile teleoperation resulted in smooth and stable control across long distances, validating the proposed system for practical use in remote or resource-constrained medical environments.

However, the architecture requires low latency and high bandwidth. Therefore, the use of a 5G network is recommended. All endpoints were interconnected through a VPN, which facilitated port forwarding requirements in ROS-based systems. The performance of 5G was found to vary depending on the position of mobile routers, particularly when human agents operated indoors. This was confirmed by RTT measurements in locations with poor signal propagation relative to the base station in Malaga. In such cases, average latency exceeded 400 ms, which compromised control precision and required lowering the robot’s speed to maintain data rates. Consequently, the deployment of 4G/5G crowd-cells—portable cellular networks [65]—is recommended in environments with limited coverage, such as those encountered in search and rescue (SAR) operations.

As part of future developments, several enhancements are envisioned to increase system robustness and usability. One key improvement involves integrating real-time transmission status indicators into the operator interface. This feature will allow users to monitor communication quality during teleoperation including latency, jitter, and packet loss, providing valuable feedback for decision, making in critical medical scenarios.

Another promising direction is the implementation of synchronous ROS services to support coordinated runtime operations. For instance, a ROS service could be exposed by the robot controller node to notify precisely when motion begins. This would allow distributed monitoring nodes to initiate latency measurements in a synchronized fashion, enabling systematic RTT analysis across geographically distributed components. In parallel, remote or local human operators could explicitly invoke ROS services to start or end teleoperation sessions through a mobile or GUI-based interface. This mechanism would add an additional layer of human authorization before enabling motion on the physical robot, reinforcing the system’s safety model, where MQTT commands are first interpreted within the DT and only executed on the robot after validation through the ROS layer.

Additionally, a latency analysis of the video streams displayed on the smartphone interface is planned. This includes evaluating the delays associated with the RGB camera feed, the ultrasound video stream, and DT visualization. These measurements will be incorporated directly into the Android application, and a dedicated “Statistics” tab will be added to present this information in real time, further improving operator awareness and system transparency. To optimize bandwidth usage in mobile environments, both the RGB and ultrasound streams received by the smartphone will be compressed using high-efficiency video codecs, such as H.265/HEVC, which offer significant data rate reductions without compromising image quality. This strategy aims to support continuous, low-latency video reception even in scenarios with constrained connectivity.

Looking forward, future work will expand the SAR-IoCA implementation by integrating a mobile manipulator, a DT server, and an MQTT–ROS bridge. The system is expected to scale to multiple use cases within the IoCA framework and support teleoperation over low-latency 5G/6G networks. The integration of location-aware services based on ROS 2, as well as imitation learning techniques for robotic manipulators, is also foreseen. Among the potential application domains are remote inspection, remote gripping or assembly in hazardous environments, telemedicine procedures, and SAR operations in catastrophic scenarios.

Furthermore, the use of a Real-Time Streaming Protocol (RTSP) server is being evaluated to acquire high-quality ultrasound images while reducing the computational load on the mobile router in field conditions. Preliminary tests indicate that this server may introduce up to two seconds of latency—unacceptable for teleoperation but potentially viable for near-real-time remote diagnostics in clinical centers. In such contexts, human agents located at MEC facilities could apply AI algorithms for illness detection. Additionally, improvements to the Human–Machine Interface (HMI) are envisioned, including real-time visualization of diagnostic insights or alerts generated by multi-agent Large Language Models (LLMs). These agents could assist the remote operator by interpreting sensor data, highlighting anomalies, or suggesting relevant actions, thereby supporting more informed and efficient clinical decision-making.

Beyond the current strategy of force threshold-based warnings for excessive contact, future versions of the system could incorporate impedance control techniques at the robot level. This would allow the manipulator to adapt its stiffness and damping in response to external forces, reducing the likelihood of excessive pressure on the subject’s body during contact. Implementing impedance control locally at the end-effector—based on real-time force sensor feedback—would complement the existing warning system by introducing proactive compliance behaviors, ultimately improving safety and enhancing the sense of natural interaction during both autonomous and teleoperated tasks.

Lastly, the application of machine learning-based data mining techniques should be explored to uncover operational patterns, detect anomalies, predict usage trends, and optimize resource allocation across distributed teleoperation systems. Future work should also prioritize the validation and refinement of the proposed performance metrics—particularly their weighting parameters—through systematic sensitivity analysis and consultation with clinical experts. Finally, regulatory certification of the robotic ultrasound manipulator will be essential before the system can be deployed in clinical environments.

## Figures and Tables

**Table 1:** Results for some LTs over the phantom.

Magnitude	LT1	LT2	LT3	LT4
teleoperation time ($t_{t}$) [s]	180.67	178.72	132.43	130.46
Body contact time (τ) [s]	55.88	44.51	45.87	51.22
Contact time ratio (ψ)	0.31	0.25	0.35	0.39
Dangerous warnings ($w_{R}$)	6	2	8	0
Caution warnings ($w_{Y}$)	15	6	14	4
Performance index (φ) [%]	57.02	52.62	62.07	88.66

**Table 2:** Results for remote teleoperation trials over the subject’s thigh.

Magnitude	RT1	RT2	RT3
teleoperation Time (t) [s]	135.42	152.01	190.06
Body contact time (τ) [s]	42.26	52.39	66.02
Body time ratio (ψ)	0.31	0.34	0.35
Dangerous warnings ($w_{R}$)	0	0	1
Caution warnings ($w_{Y}$)	0	2	5
Performance index (φ) [%]	71.77	78.51	76.44

## Data Availability

The original contributions presented in this study are included in the article. Further inquiries can be directed to the corresponding author.
